# Primary Renal Lymphoma Presenting as End-Stage Renal Disease

**DOI:** 10.1155/2017/9210648

**Published:** 2017-10-02

**Authors:** Lavjay Butani, Jonathan Ducore

**Affiliations:** ^1^Section of Pediatric Nephrology, Department of Pediatrics, University of California Davis Medical Center, Sacramento, CA, USA; ^2^Section of Pediatric Hematology and Oncology, Department of Pediatrics, University of California Davis Medical Center, Sacramento, CA, USA

## Abstract

Primary renal lymphoma is a rare entity, even more so in children. Children with primary renal lymphoma present with variable clinical features such as constitutional signs and symptoms, acute kidney injury, palpable abdominal masses, and gross hematuria. Herein we report a child who presented with seemingly advanced chronic kidney disease and was eventually diagnosed with primary lymphoma. He responded well to intensive chemotherapy and recovered renal function, although he was left with some functional limitations as a consequence of his treatment regimen. Our report highlights the importance of keeping neoplastic conditions under consideration when taking care of children with severe kidney disease of unclear etiology.

## 1. Introduction

Registry data (https://web.emmes.com/study/ped/annlrept/annualrept2014.pdf) indicate that as many as 6% of all children who receive a renal transplant (Tx) present with end-stage renal disease (ESRD) of unknown etiology. This has implications both for pre- and post-Tx management, especially since most such children have an underlying glomerular disease which can recur after the Tx [1]. Although children with renal cancers can develop chronic kidney disease (CKD) at long-term follow-up, presumably as a consequence of the treatment regimen [2], neoplastic conditions have not been reported, to our knowledge, as a cause of CKD at the time of initial presentation. We are reporting a child who presented with what appeared to be advanced CKD, attributed to an obstructive uropathy and for which maintenance hemodialysis was initiated. While undergoing evaluation for a renal Tx, he was diagnosed as having primary renal lymphoma (PRL) as a cause of his kidney disease and following initiation of chemotherapy his renal function dramatically improved such that he was able to come off dialysis and regain normal renal function. With this report, we would like to highlight PRL, a rare disease entity, as a possible cause of severe acute kidney injury and one that must be kept into consideration when the clinical picture is unclear.

## 2. Case Report

A 12-year-old boy was admitted with a 2-month history of progressively worsening fatigue. He had been noted to be anemic a month prior to his hospitalization, with hemoglobin of 7.9 g/dL and was started on oral iron by his primary care pediatrician. At his outpatient follow-up visit, because of persistence of his symptoms, repeat laboratory testing was performed and showed his hemoglobin had decreased to 7.3 g/dL. His serum potassium was 7 mEq/L, serum bicarbonate was low at 15 mEq/L, and serum creatinine was elevated at 8.7 mg/dL (estimated glomerular filtration rate [eGFR] 11 mL/min/1.73 m^2^). He was admitted to the pediatric intensive care unit via the emergency room where he received furosemide and sodium polystyrene. Studies to evaluate his renal disease included a renal ultrasound which revealed normal sized kidneys with bilateral grade 2 hydronephrosis without dilated ureters and with a distended, but thin-walled bladder; a voiding cystourethrogram showed no reflux and a normal urethra, but significant after void residual. Other studies included serum complements C3 and C4, both of which were normal, hepatitis B and hepatitis C and HIV serologies that were all negative, and an antinuclear antibody that was also negative. His urinalysis had a low specific gravity of 1.006 with no blood or protein. He was urgently started on hemodialysis because of hyperkalemia and was maintained on outpatient hemodialysis three times a week at discharge, with a presumptive diagnosis of advanced CKD likely from a urologic cause. His past medical history was uneventful with normal development and growth, and there were no documented urinary tract infections, unexplained febrile illnesses, or urinary complaints. For further evaluation of his renal disease and in preparation for a renal Tx, he underwent urodynamic studies which showed a normally compliant low-pressure bladder with complete bladder emptying. Even though he only had moderate hydronephrosis on ultrasound, due to the severity of his kidney disease, he underwent bilateral retrograde pyelograms which showed that both proximal ureters were somewhat medially deviated; the left proximal ureter had a 1 cm long narrow segment and the right proximal ureter had a 3 cm long narrowing. Double J ureteral stents were placed on both sides. Following stenting his urine output increased, and his renal function improved but only minimally, to a serum creatinine of 5 mg/dl (eGFR 19 mL/min/1.73 m^2^). Due to a concern that the narrowing was from extrinsic ureteric compression, abdominal and pelvic CT scans were performed. On these scans, performed about 2 months after his initial renal ultrasound, both kidneys were noted to be very large and almost entirely replaced with nonenhancing nodules throughout the parenchyma. Mild bilateral hydronephrosis was again noted. Also noted were multiple mixed sclerotic-lytic lesions in the L2 to L5 vertebral bodies; his serum calcium was normal. He was readmitted to the hospital for further management and underwent a percutaneous renal biopsy which established the diagnosis of diffuse large B-cell lymphoma. The biopsy tissue showed parenchyma infiltrated by large atypical pleomorphic lymphoid cells with prominent nucleoli and that stained positively with CD79A and CD 20. FISH probe for MYC rearrangement was negative. His bone marrow was normal. A whole body PET scan showed findings consistent with multifocal, infiltrative renal lymphoma associated with multifocal hypermetabolic lymphoma deposits in the axial and appendicular skeleton and retroperitoneal lymph nodes. With a diagnosis of PRL, he was started on chemotherapy with vincristine, cyclophosphamide, prednisone, and intrathecal methotrexate. Following a lack of response to therapy, he was transitioned to a more aggressive protocol, consisting of additional doses of vincristine, prednisone, methotrexate, doxorubicin, etoposide, and intrathecal methotrexate. Over time, his renal function progressively improved such that he was able to come off hemodialysis, and at discharge from the hospital, he had a serum creatinine of 1.7 mg/dl (eGFR 58 mL/min/1.73 m^2^). His subsequent clinical course was complicated by episodes of fever and neutropenia, typhlitis, and neurotoxicity from etoposide and vincristine needing extensive rehabilitation. At his last clinic visit, 5.5 years after diagnosis and 4 years after completing chemotherapy, his PET scan showed no hypermetabolic lesions to suggest active disease, his blood pressure was normal, and his serum creatinine was stable at 1.2 mg/dL (eGFR 94 mL/min/1.73 m^2^). On his most recent ultrasound, he had mild left hydronephrosis with a significant reduction in renomegaly; his ureteral stents had been removed in the interim. He was in school performing well academically and using a walker for ambulation due to his residual neurologic injury.

## 3. Discussion

PRL is a very rare disease entity with a reported incidence of 0.7% of all extranodal lymphomas, is usually of B-cell origin, as in our patient, and is typically described in older adults [3]. The tumor has been hypothesized to originate from mucosa-associated renal lymphoid tissue or from lymphatics in the renal capsule [3, 4]. Clinical presentations include palpable renal masses [3], hematuria, constitutional symptoms, hypertension [4], and acute kidney injury [5]. Due to the rarity of this condition, diagnosis is often delayed and limited therapeutic guidelines are available. Prognosis is reported to be poor with 25% 1-year survival [6]. Our patient is unique in that he presented with severe kidney disease and the consequent anemia, as his initial presenting manifestation, and was presumed to have CKD. The etiology of the kidney disease was presumably mainly from infiltration of the renal parenchyma by the malignant cells with an added component of extrinsic compression of the proximal ureters by the intraparenchymal masses. This is supported by the lack of a significant decline in his serum creatinine after ureteral stenting and the subsequent prompt improvement of renal function following start of chemotherapy.

With this report, we highlight PRL as a possible cause of acute kidney injury in children and one that should be thought of when no clear identifiable etiology for the kidney disease can be established. While rare, the implications of missing the diagnosis are significant as are the potential benefits of early diagnosis. In the setting of only mild hydronephrosis and the absence of atrophic kidneys, potentially reversible causes of kidney disease must be sought after in children with seemingly advanced CKD.

## References

[1] Butani L. (2006). Children presenting with end-stage renal disease of unexplained etiology: Implications for disease recurrence after transplantation. *Pediatric Transplantation*.

[2] Stefanowicz J., Kosiak M., Romanowicz G., Owczuk R., Adamkiewicz-Drozyńska E., Balcerska A. (2011). Glomerular filtration rate and prevalence of chronic kidney disease in Wilms' tumour survivors. *Pediatric Nephrology*.

[3] Dash S. C., Purohit K., Mohanty S. K., Dinda A. K. (2011). An unusual case of bilateral renal enlargement due to primary renal lymphoma. *Indian Journal of Nephrology*.

[4] Becker A. M., Bowers D. C., Margraf L. R., Emmons J., Baum M. (2007). Primary renal lymphoma presenting with hypertension. *Pediatric Blood and Cancer*.

[5] Ozaltin F., Yalçin B., Orhan D. (2004). An unusual cause of acute renal failure: Renal lymphoma. *Pediatric Nephrology*.

[6] Kandel L. B., McCullough D. L., Harrison L. H., Woodruff R. D., Ahl E. T., Munitz H. A. (1987). Primary renal lymphoma. Does it exist?. *Cancer*.

